# The head and neck cancer cell oncogenome: a platform for the development of precision molecular therapies

**DOI:** 10.18632/oncotarget.2417

**Published:** 2014-11-04

**Authors:** Daniel Martin, Martin C. Abba, Alfredo A. Molinolo, Lynn Vitale-Cross, Zhiyong Wang, Moraima Zaida, Naomi C. Delic, Yardena Samuels, J. Guy Lyons, J. Silvio Gutkind

**Affiliations:** ^1^ Oral and Pharyngeal Cancer Branch, National Institutes of Health, Bethesda, USA; ^2^ CINIBA, Facultad de Ciencias Médicas, Universidad Nacional de La Plata, La Plata, Argentina; ^3^ Department of Molecular Cell Biology, The Weizmann Institute of Science, Rehovot, Israel; ^4^ Dermatology, University of Sydney, Camperdown, Australia; ^5^ Cancer Services, Royal Prince Alfred Hospital, Camperdown, Australia

**Keywords:** HNSCC, Sequencing, Exome, RNAseq, Cancer

## Abstract

The recent elucidation of the genomic landscape of head and neck squamous cell carcinoma (HNSCC) has provided a unique opportunity to develop selective cancer treatment options. These efforts will require the establishment of relevant HNSCC models for preclinical testing. Here, we performed full exome and transcriptome sequencing of a large panel of HNSCC-derived cells from different anatomical locations and human papillomavirus (HPV) infection status. These cells exhibit typical mutations in *TP53*, *FAT1*, *CDK2NA*, *CASP8*, and *NOTCH1*, and copy number variations (CNVs) and mutations in *PIK3CA*, *HRAS*, and *PTEN* that reflect the widespread activation of the PI3K-mTOR pathway. *SMAD4* alterations were observed that may explain the decreased tumor suppressive effect of TGF-β in HNSCC. Surprisingly, we identified HPV^+^ HNSCC cells harboring *TP53* mutations, and documented aberrant TP53 expression in a subset of HPV^+^ HNSCC cases. This analysis also revealed that most HNSCC cells harbor multiple mutations and CNVs in epigenetic modifiers (e.g., EP300, CREBP, MLL1, MLL2, MLL3, KDM6A, and KDM6B) that may contribute to HNSCC initiation and progression. These genetically-defined experimental HNSCC cellular systems, together with the identification of novel actionable molecular targets, may now facilitate the pre-clinical evaluation of emerging therapeutic agents in tumors exhibiting each precise genomic alteration.

## INTRODUCTION

HNSCC is the sixth most common cancer worldwide, with more than 500,000 new cases each year, of which only 40–50% will survive for 5 years. Over 42,000 new cases of HNSCC are predicted to be diagnosed and 8,300 deaths to occur in 2014 from this disease in the United States alone [1]. Exposure to tobacco carcinogens combined with alcohol is a major risk factor in Western countries, while betel quid and areca nut chewing are risk factors commonly found in the south Asian region [2]. In the past few decades, sexually transmitted infection with high risk human papillomaviruses (HPV) has also emerged as a major risk factor, particularly affecting a younger population [3, 4]. Recent advances in HNSCC treatment have improved the quality of life and life expectancy of HNSCC patients if this disease is diagnosed at early stages [5]. However, the overall survival of HNSCC patients, the majority of which are diagnosed at advanced stages, has only improved marginally over the past 30 years. Currently, the most common HNSCC therapeutic modalities include the use of nonselective treatments (surgery, radiation and chemotherapy) with very high systemic toxicities and associated morbidity and mortality. The development of more selective cancer treatment options for HNSCC will benefit from the complete understanding of the molecular mechanisms -and the underlying genetic alterations- in HNSCC carcinogenesis in order to identify actionable targets of therapeutic value.

In this regard, the development of novel therapeutic modalities invariably involves the initial evaluation in preclinical cancer models; hence the availability of relevant and well characterized biological systems is invaluable. For HNSCC, most preclinical studies have traditionally involved the use of widely available human HNSCC cell lines that form tumors in immunocompromised mice, some of which can also recapitulate the ability of HNSCC to invade loco-regional lymph nodes and even develop distant metastasis [6–8]. However, the lack of information regarding the primary molecular alterations in these cell lines has hampered the possibility of interpreting the emerging pre-clinical activity of recently developed therapeutic agents with the underlying mechanisms driving HNSCC progression. This is particularly relevant in the new era of precision medicine, in which the genetic alterations in each HNSCC lesions can now be assessed in a clinically relevant setting. Thus, building on prior multi institutional cancer sequencing efforts [9–12], we have now characterized the genetic alterations and expressed messages of a large collection of representative HNSCC lines, including normal immortalized oral keratinocytes as well as cell lines derived from HPV^−^ and HPV^+^ oral tumor lesions. This HNSCC panel includes cell lines harboring the most frequent HNSCC alterations, which may now provide a valuable tool for the future development and evaluation of molecular-guided therapeutic options for HNSCC.

## RESULTS

We selected a group of HNSCC cells (herein referred as oral and pharyngeal cancer (OPC)-22 panel) and subjected them to a thorough characterization involving short tandem repeat (STR) analysis, whole exome capture sequencing, and mRNA sequencing. Our primary goal was to develop a HNSCC cell line panel resembling the breadth and complexity of genetic alterations found in HNSCC at large. To preserve the genetic diversity found in HNSCC, we included HNSCC lines developed in cancer research centers covering distinct geographic locations, aimed at minimizing potential haplotype biases. We also included a non-transformed, spontaneously immortalized normal oral keratinocyte (NOKSI) cell line [13] which could provide additional insight into the molecular mechanisms involved in immortalization and premalignancy. The initial characterization of this panel involved STR profiling (Supplemental Table 1). We confirmed the correct identity of previously reported cell lines (CAL27, CAL33, Detroit 562, UM-SCC-47, SCC-25, SCC-9, UM-SCC-11B and UM-SCC-17B) [6], while the information generated about previously unreported HNSCC cell lines should serve as a *bona fide* reference for future studies. Available clinical information on the OPC-22 cell lines compiled from different sources is provided in Supplemental Table 2.

The use of established cancer cell lines prevents somatic mutation calling by comparing the sequence information with respect to matched normal DNA, as the latter is usually unavailable. Thus, to identify putative somatic mutations in the HNSCC panel we used a variation of a production-level filtering strategy [14] involving the rejection of variants present in the dataset derived from the NIH/NHLBI ESP6500 project (variant frequency not equal to 0), and the rejection of variants present in more than 15% of the lines (3 cell lines) as putative uncharacterized SNPs, unless they were present in the COSMIC v64 database [16]. The latter was used to salvage true highly frequent mutations in cancer. For a comprehensive list of mutations see the Supplemental Data File 1.

**Figure 1:**
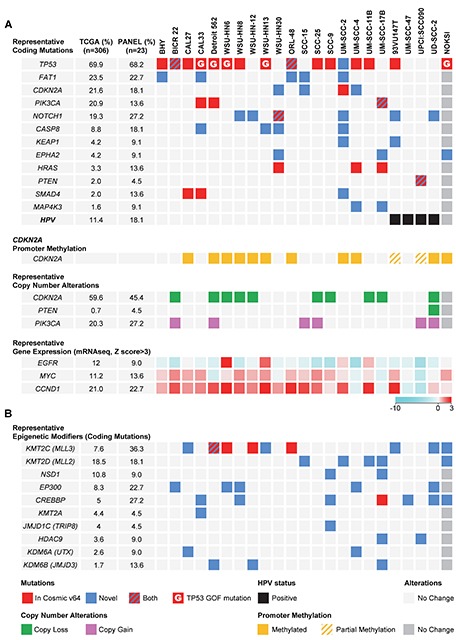
The most frequent alterations in representative HNSCC-derived cells **(A)** Top panel, graphical matrix representation of the individual mutations in 22 HNSCC cells and a normal spontaneously immortalized oral keratinocyte line (NOKSI, dark grey to denote the exclusion from the OPC-22 panel). Individual genes are represented in rows and cell lines in columns. In some cases more than one mutation per gene is present. For a comprehensive list see Supplemental Data File 1. The HPV status of each HNSCC-derived cell is represented in the bottom row. Second panel, PCR based promoter methylation analysis of the *CDKN2A* gene. Third panel, representative per-gene copy number variations as derived from comparison of each cell line to a computed pseudo-normal. Fourth panel, representative gene expression levels as determined by RNAseq data. Color code represents a log2 transformed fold expression normalized to the median of all samples. **(B)** Mutations in genes encoding histone modifying enzymes. Red square, mutation described in the COSMIC v64 database. Blue square, novel mutation. Red/Blue square, two or more mutations in a gene, one being novel and the other present on the COSMIC v64 database. Red square with inlay G, *TP53* mutation present in the COSMIC v64 database defined as Gain-Of-Function. Green square, Gene copy loss, representing both hetero and homozygous deletions. Pink square, Gene copy gain, representing both copy gain and gene amplification. Black square, HPV status. Yellow square, *CDKN2A* promoter region methylation, no unmethylated product was detected. Yellow/Gray, *CDKN2A* promoter methylation analysis detected both methylated and unmethylated products. Light and dark grey squares, no change.

Based on prior studies addressing the most common gene alterations in HNSCC [9, 10], we then compared their mutation frequency in the OPC-22 cell panel with respect to that found in the Cancer Genome Atlas consortium (TCGA) Head and Neck cancer provisional dataset, which currently comprises 306 HNSCC tumor samples (accessed through the cBioPortal, http://www.cbioportal.org). Interestingly, the frequency of mutations in the OPC-22 panel closely resembled that of the TCGA (Figure 1A). *TP53* is the most frequently mutated gene both in HNSCC (69.9%) and the OPC-22 cell set (68.2%). Most of these alterations are present in the COSMIC database, while some additional novel *TP53* mutations were detected in BICR22, WSU-HN12 and UM-SCC-2 cells, which are predicted to be deleterious (see Supplemental Table 3). On the other hand, *TP53* gain of function (GOF) mutations H179L, V173L and R175H [17, 18] were detected in HN6, HN13 and CAL33 and Detroit 562 respectively.

In agreement with available information [19–21], activation of the PI3K/Akt/mTOR pathway is emerging as a leading oncogenic mechanism in HNSCC. The OPC-22 panel nicely recapitulated the common occurrence of *PIK3CA* mutations (Figure 1A, upper panel) as well as *PIK3CA* gene amplification as depicted by copy number variation (CNV) analysis (Supplemental data file 2). In total, 9 out of 22 cell lines displayed either activating *PIK3CA* mutations or gene amplification, the latter noticeably overrepresented in HPV^+^ HNSCC lines (2 out of 4 HNSCC cell lines show amplification). Moreover, mutation of the *PTEN* phosphatase occurred in UPCI:SCC090 while homozygous deletion of *PTEN* was detected in UD-SCC-2, both derived from HPV^+^ HNSCCs. *HRAS* activating mutations were detected at low frequency in HNSCC (3.3%), although this percentage was slightly higher (13.6%) in the OPC-22 set.

The protocadherin FAT1 is the second most mutated gene in HNSCC. In our panel this molecule was mutated in 5 of the cell lines (Figure 1A) and deleted in 2 (SCC- 25 and SCC-9, see Supplemental Data File 2). FAT1 functions in HNSCC are not well understood, but it has been recently associated with the regulation of β-catenin complexes, therefore contributing to a migratory and invasive phenotype when its function is compromised [22, 23]. Another highly altered gene is the cell cycle inhibitor p16 (*CDKN2A*), which presented either somatic mutations, extensive promoter methylation, as judged by PCR on bisulfite-treated genomic DNA (Figure 1A, second panel), or gene copy loss (Figure 1A, third panel). The NOTCH protein family has been recently identified as frequently mutated in HNSCC, and the functional implications of NOTCH alterations are now under investigation [9, 10, 24]. Loss of function alterations have been reported for all the family members, but they are more prominent in NOTCH1 (19%) and are similarly well represented in the OPC-22 panel (27.2%). The receptor associated caspase CASP8 is also frequently mutated in HNSCC [12], and its coding sequence is altered in 18% of the cell lines. In addition to its role in HNSCC development by preventing TNF-induced apoptosis [25], specific cancer-associated missense mutations have been recently shown to induce NF-κB activation [26], a well-established pro-oncogenic player in HNSCC [27].

Less explored mutations and genomic alterations were also identified in the OPC-22 panel. The antioxidant response master regulator NFE2L2 (NRF2) transcription factor [28] is frequently amplified and mutated in HNSCC (5.6%). Mutations in specific residues impair its interaction with the endogenous inhibitor and redox sensor KEAP1, which is also frequently mutated in HNSCC, ultimately leading to increased transcriptional activity and resistance to oxidative stress [29]. While no NFE2L2 mutations were identified in the OPC-22 panel, two HNSCC cell lines contained KEAP1 mutations of unknown function. The tyrosine kinase EPHA2 is mutated in a small number of HNSCCs (4%). Its role in HNSCC development and progression is not well understood [30], but its mutation profile, including a high fraction of nonsense and frameshift alterations suggest a tumor suppressive role. Interestingly, two cell lines in the OPC-22 panel and NOKSI contain mutations in the gene for this molecule, and include a frameshift (P212fs in UM-SCC-17B) and a nonsense mutation (W456X in NOKSI).

Of particular interest, *SMAD4* was mutated in 3 of our cells lines, which is higher than expected based on the actual rate on the TCGA dataset. Although it is rarely mutated in HNSCC, the SMAD4 protein was found to be frequently lost (see below). Finally, we identified mutations in MAP4K3 (also called GLK), a kinase that has been recently identified as a component of the mTORC1 complex and as an upstream regulator of the JNK pathway [31]. MAP4K3 is not frequently mutated in HNSCC (1.6%), but the mutation found in UM-SCC-17B (V322M) is identical to that identified in three cases of the Cancer Cell Line Encyclopedia (CCLE). Because its involvement in the regulation of mTOR by amino acids [31], MAP4K3 has a potential role in HNSCC development.

We were unable to detect activating mutations of EGFR, but identified strong overexpression of its mRNA in HN6 and HN13 in (Figure 1A fourth panel). Likewise, overexpression of Cyclin D1 (*CCND1*) was very prominent among all the lines, except in the HPV^+^ group. Interestingly, *MYC* expression closely resembled that of *CCDN1*, perhaps indicating that the concomitant overexpression of *CCDN1* and *MYC* contributes to HNSCC progression in the absence of HPV-specific oncogenic mechanism that may bypass this requirement.

Of note, multiple cell lines did not exhibit molecular alterations in readily identifiable oncogenic drivers, which is also apparent in many HNSCC cases analyzed in TCGA, and in agreement with other reports [9, 10]. In search for candidate transforming events, we noticed that two histone methyltransferases, NSD1 and KMT2D (MLL2) belonging to the family of epigenetic regulators rank amongst the most frequently mutated genes in HNSCC. In fact, when we studied the presence of mutations in molecules involved in epigenetic gene expression control (Figure 1B), we found a surprisingly high incidence of alterations throughout the HNSCC panel. Only 4 out of the 22 cells lines showed no alterations in epigenetic modifying enzymes, while the rest contained at least one if not multiple overlapping alterations in key histone modifiers. In this regard it is interesting to note that most alterations are predicted to be deleterious mutations, and therefore interfering with epigenetic regulation. Despite the potential functional redundancy of the epigenetic regulating machinery, we noticed that very specific functions seem to be highly represented. For example, the EP300 and CREBBP histone acetyltransferases, which play a crucial role in the activation of gene expression, are frequently altered (12.9%) in a non-overlapping fashion in all cases analyzed in TCGA. These alterations seem to be much more prominent in HNSCC lines, where we detected alteration in either *EP300* or *CREBBP* in almost 50% of the cases. While the reason for this increased representation of *EP300*/*CREBBP* mutations in HNSCC cells lines is unclear, it is possible that these alterations diminish differentiation and thereby enables the establishment of HNSCC cell cultures.

Another interesting example is the frequent alteration of KDM6A (UTX) and KDM6B (JMJD3). These two molecules are the only enzymes displaying H3K27 di- and tri-demethylase activity and are strictly required for the reactivation of genes that have been epigenetically silenced. These molecules are mutated in 2.6 and 1.7% of HNSCC cases deposited in TCGA, respectively, in a mutually exclusive fashion. When including alteration due to copy number variation (Supplemental Table 4), we observed that KDM6A is homozygously deleted in an additional 5% of the tumors in TCGA. However, due to the location of KDM6A in the X chromosome this gene product is highly sensitive to hemizygotic loss, an event that is detected in 29.5% of HNSCC cases in TCGA. Considering mutations, hemi- and homozygotic loss of KDM6A elevates the number of altered HNSCC cases to 36.7%, highlighting the prominent alteration of this molecule in HNSCC. Moreover, KDM6B is also frequently hemizygously deleted (24.8%) and in over 10% of the cases both enzymes are hemizygously deleted, and therefore total H3K27 demethylase gene dosage is effectively halved.

**Figure 2:**
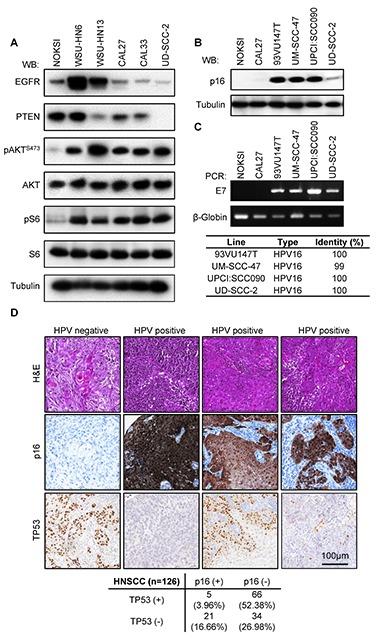
Exome sequencing data validation **(A)** Biochemical characterization of alterations in PI3K-mTOR predicted by whole exome and RNAseq data for representative cell lines. Exponentially growing cultures were serum starved overnight and then lysed. A representative Western blot is shown for each indicated protein and phospho-protein. **(B)** Analysis of *CDKN2A* (p16) levels in HPV+ cell lines. **(C)** Upper panel, status of HPV infection by LCR-E7 PCR. HPV type identities were determined by Sanger sequencing of the LCR-E7 PCR amplicons. **(D)** A representative example of a cohort of 126 HNSCC tumors for which HPV status and *TP53* mutation was evaluated as judged by immunohistochemical staining. A proportion of HPV+ (p16+) cases displays TP53 immunoreactivity, indicating the accumulation of *TP53* mutant forms. TP53 staining in HPV+ samples varied in proportion and intensity. A quantification of the results of this study is presented in the lower panel.

We next sought to confirm the predictive value of exome and mRNA sequencing by analyzing relevant alterations in our panel. mRNA sequencing revealed high *EGFR* expression levels on WSU-HN6 and WSU-HN13 cell lines, which was readily observed as increased protein levels by Western blot in these two particular cells lines (Figure 2A). Moreover, copy number variation analysis derived from exome sequencing data detected the homozygous deletion of the *PTEN* phosphatase in UD-SCC-2 cells in agreement with the complete absence of PTEN protein expression. These two events can potentially lead to the activation of the mTOR kinase which itself is a widespread event in HNSCC tissues and cell lines [20, 21, 32]. As shown in Figure 2A, phosphorylation of the mTOR targets AKT^S473^ and the pS6 is prominent in all HNSCC cell lines.

Persistent HPV infection with high risk HPV types, primarily HPV16, is emerging as a leading risk factor for the development of HNSCC [3, 33]. Four of the cell lines in the OPC-22 panel have been previously reported to be HPV^+^ [6, 34, 35], namely 93VU147T, UM-SCC-47, UPCI:SCC090 and UD-SCC-2. Expression of the p16 product of the *CDKN2A* gene, an inhibitor of the G1/S regulator CyclinD-cyclin dependent kinase (CDK)4/CDK6, has traditionally been used as a surrogate marker of HPV infection [13, 36]. Under normal conditions, p16 expression is epigenetically silenced [37]. Due to the dysregulation of the RB tumor suppressor by binding to the HPV-encoded E7 oncoprotein, RB no longer needs to be inactivated by CDK4/6-mediated phosphorylation, and this leads to cell cycle progression and cell proliferation despite the massive accumulation of cell cycle inhibitors such as p16 [38]. Interestingly, we observed elevated expression of p16^*INK4A*^ by Western blot in all the HPV^+^ cells (Figure 2B). Moreover, PCR amplification and sequencing of genomic DNA revealed the presence of E7-like product in all the HPV^+^ HNSCC lines (Figure 2C). Sanger sequencing of the resulting amplicons confirmed the presence of HPV16 infection in every case (Figure 2C, lower panel). This is in agreement with the notion that HPV16 is the most common high-risk HPV type in HNSCC [3].

Due to the molecular function of the HPV E6 oncoprotein *TP53* alterations are expected to be absent in HPV positive HNSCCs as depicted by prior studies [9, 10]. However, one of the HPV positive lines, 93VU147T, exhibited a *TP53* mutation (L257R) predicted to be deleterious. Therefore we wanted to address if the presence of *TP53* alterations in HPV-related HNSCCs is a more common event in HNSCC than previously recognized. We studied the co-expression of TP53 and p16^INK4A^ proteins in a cohort of 126 cases of HNSCC. Detection of TP53 by immunohistochemistry is frequently used as an indication of mutated or inactive TP53 [39]. As shown in Figure 2D, the majority of the HNSCC samples show only TP53 staining (52.38%), and a smaller number showed only p16 staining (16.66%). However, 3.17% of the samples showed a co-staining of p16 and TP53. In this regard, we observed two distinct patterns of TP53 staining in p16 positive tumors. About half of the HPV^+^ samples displayed extensive TP53 staining while the other displayed small clusters of TP53 positive tumor cells, probably representing small clonal populations within the tumor.

**Figure 3:**
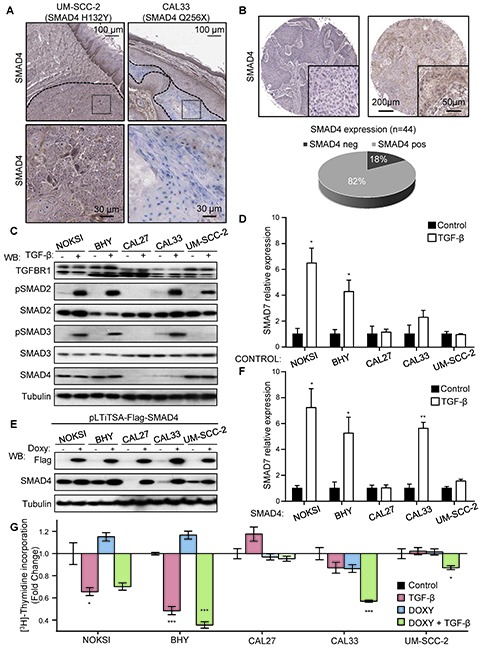
Aberrant TGF-β signaling in HNSCC and the OPC-22 panel **(A)** Sequencing data identified the presence of mutations in CAL33 and UM-SCC-2 as indicated. A representative SMAD4 staining on tumor xenografts with an antibody raised against the C-terminus of SMAD4 detected expression (brown) in UM-SCC-2 tumors and mouse stroma, while CAL33 tumors were negative. Tumor areas are delimited by a dashed line. Dotted area insets are shown at higher magnification in the corresponding lower panels. **(B)** Analysis of a cohort of 44 HNSCC cases stained for SMAD4. A representative negative (left) and positive (right) case is shown. Whole cohort quantification is shown in the lower panel. **(C)** Analysis of the TGF-β signaling in select HNSCC-derived lines. Cells were cultured under exponential growing conditions and then serum starved overnight. Cells were stimulated with vehicle (-) or 100 ng/ml TGF-β (+) for 45 minutes. Cells lysates were analyzed by Western blot for the proteins and phospho-proteins indicated in the figure. **(D)** Cells cultured as in C, were stimulated for 6h with vehicle (black bars) or 100 ng/ml TGF-β (white bars) and then RNA was extracted. SMAD7 expression was determined by qPCR. n=4, *, p≤0.05 for TGF-β different from Control. **(E)** A doxycycline inducible Flag-SMAD4 WT-IRES-GFP lentivirus was engineered and used to infect HNSCC as indicated. The percentage of SMAD4 WT expressing cells was determined to be over 70% in each case by GFP analysis. Cells cultured in the presence of 1 μg/ml doxycycline for 18h were lysed and analyzed by Western blot. **(F)** Cells in exponentially growing conditions were serum starved 12h in the presence of 1 μg/ml doxycycline and then stimulated for 6h with vehicle (black bars) or 100 ng/ml TGF-β (white bars). RNA was then extracted and *SMAD7* expression levels determined by qPCR. n=4, *, p≤0.05, **, p≤0.01. **(G)** Cell proliferation assay by [^3^H]-thymidine incorporation. Exponentially growing cultures in the presence or absence of doxyclycline as indicated for 24h. Cells were then serum starved and treated with TGF-β while maintaining doxycycline treatment. [^3^H]-Thymidine (1μCi) was added to the cultures 4 h before the end of the treatment (total treatment time 24h). n=4, *, p≤0.05, ***, p≤0.001

As described above, another frequent alteration detected in the HNSCC cell panel was the presence of inactivating mutations of SMAD4. This co-SMAD is strictly required for proper receptor-SMAD (R-SMAD) signaling downstream of TGF-β receptor family, including TGFBR1/2 as well as the bone morphogenetic protein (BMP) and activin receptors [40]. Two cell lines in our panel, CAL27 and CAL33, harbor truncating mutations of SMAD4, while UM-SCC-2 displayed a H132Y mutation predicted to be deleterious (PROVEAN score −5.490). While the frequency of SMAD4 mutations reported in the TCGA dataset is low (2%), this gene is very frequently hetero and homozygously deleted (48.7 and 4.6%, respectively). We validated our sequencing observations by studying the expression of SMAD4 in CAL33 and UM-SCC-2 cells, exhibiting a truncated and mutant SMAD4, respectively, using an antibody that recognizes the c-terminus of SMAD4. We were able to detect cytoplasmic and perinuclear SMAD4 staining in UM-SCC-2 xenografts, but failed to detect SMAD4 expression in CAL33 tumors while strongly reacting with the mouse stroma (Figure 3A). These findings prompted us to screen a HNSCC tissue array for SMAD4 expression by immunohistochemistry in order to better assess the true frequency of SMAD4 alterations in HNSCC at the protein level. We identified the absence of detectable SMAD4 expression in ~18% of the samples (n=44, Figure 3B).

Because TGF-β signaling elicits both pro-proliferative and tumor-suppressive responses depending on the biological context [40], we addressed the specific impact of TGF-β dysregulation in HNSCC in cell lines harboring SMAD4 alterations (CAL27, CAL33 and UM-SCC-2), using non-tumorigenic, spontaneously immortalized normal oral keratinocytes (NOKSI) and a SMAD4 wild type containing HNSCC line (BHY) as controls. As shown in Figure 3C, treatment with TGF-β induced the robust phosphorylation of the downstream R-SMAD SMAD2 in all cells except CAL27. Concomitant SMAD3 phosphorylation was also absent in CAL27 and UM-SCC-2 cells. Further analysis revealed that the failure to properly phosphorylate R-SMADs in CAL27 is likely due to the presence of a deleterious mutation in TGFBR1 (N45S, PROVEAN score −3.275). SMAD4 levels were very low in both CAL27 and CAL33, as expected. Despite proper activation of at least one R-SMAD in CAL33 and UM-SCC-2, TGF-β failed to induced gene expression of SMAD7, a prototypical transcriptional target of TGF-β signaling and negative regulator of the pathway (Figure 3D).

In order to address the biological role of SMAD4 inactivation in HNSCC we sought to rescue SMAD4 function by infection with an inducible lentivirus encoding a Flag-tagged wild type version of SMAD4. As show in Figure 3E, we successfully generated doxycycline-inducible SMAD4 expressing HNSCC cell lines, as reflected by Flag immunodetection. We then challenged the Flag-SMAD4 expressing cell lines with TGF-β (Figure 3F), and observed a substantial recovery of SMAD7 expression in CAL33 cells, while CAL27 and UM-SCC-2 remained insensitive. We concluded that TGFBR1 alterations in CAL27 impairs TGF-β signaling, while in the absence of other detectable abnormalities in the pathway, the SMAD4 H132Y mutation present in UM-SCC-2 likely behaves as a dominant negative protein, effectively preventing SMAD2/3-mediated transcription in this HNSCC line.

Finally, we asked whether impaired TGF-β provided any proliferative advantage in these cell lines. We conducted a cell proliferation assay in these engineered cell lines (Figure 3G) and observed that TGF-β treatment decreases the proliferation of both NOKSI and BHY cells. This antiproliferative response was absent in cells where TGF-signaling was defective (CAL27, CAL33 and UM-SCC-2). Interestingly, when SMAD4 expression was restored via induction with doxycycline, CAL33 showed a significant reduction in proliferation in response to TGF-β, while CAL27 and UM-SCC-2 remained refractory, mirroring the induction of SMAD7 expression changes observed in Figure 3F. We therefore concluded that the role of TGF-β signaling in HNSCC is antiproliferative, in agreement with previous reports [41], and that this response is dependent on proper R-SMAD and co-SMAD signaling leading to productive TGF-β gene expression regulation.

**Figure 4:**
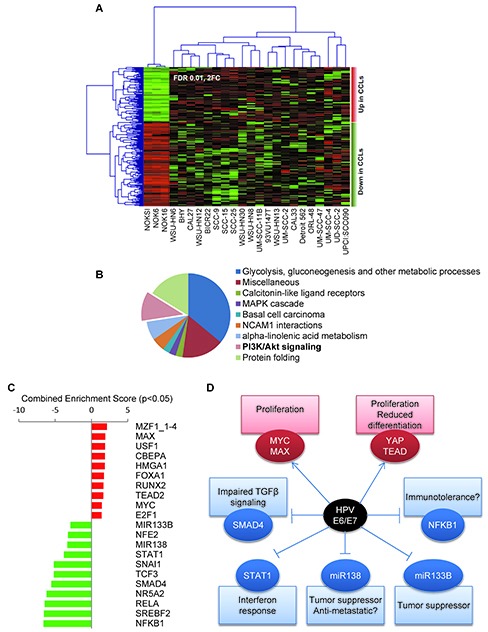
Gene expression analysis of the OPC-22 panel **(A)** Supervised clustering analysis of the OPC-22 HNSCC lines on differentially expressed genes (FDR<0.01, Fold change ≥2) when comparing HNSSC lines to normal. Two additional normal cells lines (NOK6 and NOK16) represent two additional independent isolates from the same donor and were included in this analysis for statistical purposes. UM-SCC-17B was removed from the analysis due to outlier expression profile. **(B)** Functional enrichment analysis on biological processes on differentially expressed genes using the ClueGO tool. **(C)** Genes differentially expressed between HPV+ and HPV- groups (Moderated T-test, p<0.05, fold change >±2) were analyzed by the Enrichr tool against the TRANSFAC and JASPAR position weight matrices (PWMs) for predicted transcription binding sites in their respective promoter regions. The combined score is a positive value computed by taking the log of the p-value from the Fisher's exact test and multiplying that by the z-score of the deviation from the expected rank. Enrichment scores derived from the downregulated gene list were given negative value for representation purposes. **(D)** Schematic representation of a hypothetical HPV E6/E7 interaction network leading to differential gene expression in the OPC-22 HPV+ cohort.

We next performed an analysis of the OPC-22 transcriptome in order to identify global changes in gene expression in HNSCC cells. Initial quality control identified the expression profile of UM-SCC-17B as an outlier, likely due to technical issues, and was removed from subsequent analyses. To identify the most representative differentially expressed transcripts between immortalized oral keratinocyte lines and the HNSCC cells, we employed the edgeR test as statistically supervised method (Figure 4A). This analysis revealed 230 genes differentially expressed (FC>2; FDR<0.01) of which 90 were upregulated and 140 were downregulated transcripts in HNSCC cells (Supplemental Data File 3). Functional analysis of the deregulated gene list identified statistically significant enrichment of biofunctions associated to several metabolic processes, protein folding and cell signaling related to FGFR1, PI3K/AKT and MAPK cascade pathways (Figure 4B). To identify affected transcriptional regulatory networks, we performed a transcription factor enrichment analysis from the deregulated gene list. This allowed us to identify a set of transcription factors whose activity is potentially upregulated in HNSCC cells, which included HIF1α, NFκB1 and JUN, and the CDKN2A suppressor ZBTB7A [42] (see Supplemental Data File 4).

Because HPV-associated HNSCCs represent a distinct clinical entity, we took advantage of the sequence information of the HNSCC HPV^+^ cell transcriptome to help define its unique transforming mechanisms. By performing a supervised class comparison between the HPV^+^ and HPV^−^ cells lines in the panel, we identified 109 differentially regulated genes (>2 Fold change, p<0.05, Supplemental Data File 5). Analysis of 68 upregulated genes helped identify a significant enrichment (Supplemental Data File 6) in several upstream regulators (transcription factors) including MZF1, MYC/MAX, YAP/TEAD2 and E2F1, while the downregulated genes (n=41) appeared to be enriched on transcriptional targets of the NF-κB/RELA, NR5A2, STAT1, SMAD4 and SNAIL transcription factors and MIR133B and MIR138 microRNAs (Figure 4C). A scheme representing potential biological consequences based on the predicted transcriptional events responsible for the gene expression profile of HPV^+^ cell lines is presented in Figure 4D, thus suggesting multiple unique molecular mechanisms that might underlie HPV-associated head and neck malignancies.

## DISCUSSION

The rapid progress of targeted therapies and our increased understanding of the molecular basis of HNSCC may soon enable personalized medicine approaches based on the genetic and epigenetic alterations of each tumor lesion. However, the development of new precision molecular treatment options requires the availability of suitable preclinical models. Here, we have characterized a representative panel of HNSCC cells lines reflecting the most frequent genetic alterations observed in HNSCC. This biologically relevant experimental system will be important to study the impact of specific treatment options based on each genomic alteration. In addition, many of these HNSCC cells have been used extensively; hence there is wealth of biochemical and biological data already available worldwide that can now be re-analyzed retrospectively in light of the molecular alterations underlying each HNSCC cell model.

In this regard, prior efforts have provided an initial genomic characterization of several HNSSC cell lines, some of which are also included in our study [43–45]. In the current study, all cells lines have been sequenced and analyzed using a common platform, thus minimizing methodological biases. Two other critical differences are that our current study extends the geographical diversity of HNSCC cells as they were collected from different sources around the world, and that the present HNSCC cell panel included a cohort of HPV^+^ cell lines. Indeed, given the increasing number of HPV-related HNSCC cases, with a clear tendency to increase in the future [3], the inclusion of 4 different HPV^+^ HNSCC cell lines may now help reflect the genomic and gene expression characteristics of HPV^+^-associated HNSCC. Therefore the OPC-22 panel may provide a relevant biological and molecular toolbox for the study of HNSCCs exhibiting distinct genetic alterations and HPV status.

A number of emerging themes can be derived from the systematic analysis of the OPC-22 panel. Firstly, it reinforces multiple observations that deregulation of the PI3K-mTOR pathway may represent a key oncogenic driving mechanism in HNSCC [19]. Mutations and amplification of *PIK3CA* are frequent in HNSCC, whereas *PTEN* mutations or deletions are not [9–12, 19]. It is therefore intriguing that alterations in *PIK3CA* and *PTEN* accumulate within the HPV^+^ cells lines, as reflected by the fact that we found *PTEN* mutated (UPCI:SCC090) or homozygously deleted (UD-SCC-2), and that *PIK3CA* is consistently amplified (93VU147T, UM-SCC-47, UPCI:SCC-90, UD-SCC-2). These findings suggest that robust PI3K pathway activation in a *TP53* and *RB* inactive background conferred by the function of the HPV E6/E7 oncoproteins might be sufficient to induce HNSCC tumor progression. This might have direct implications in the management of HNSCC, as *PIK3CA* and *RAS* activating mutations in HNSCC cells both confer resistance to cetuximab, an EGFR-targeted antibody commonly used as a first line treatment in HNSCC patients [46].

Another interesting observation was a broad dysregulation of the TGF-β signaling system in HNSCC. Previous reports indicated the infrequent alteration of SMAD4 in HNSCC [47], and indeed, the mutation frequency of *SMAD4* as defined by the TCGA dataset is low (2%). However, we found that additional *SMAD4* alterations such as homo- and heterozygous deletions are much more frequent (5% and 49%, respectively). Hence, loss of expression due to gene deletion is likely the most relevant mechanism leading to SMAD4 inactivation in HNSCC, while mutations leading to early termination codons as well as other inactivating and dominant negative mutations may compromise SMAD4 function in a large fraction of HNSCC cases. Based on our analysis of HNSCC cell lines and the information available at the TCGA, SMAD4 alterations disrupting TGF-β signaling are likely close to 20%. Indeed, in our analysis of HNSCC tissue arrays, SMAD4 expression was undetectable in 18% percent of the cases. We can also speculate that because of the crucial role of SMAD4 in TGF-β induced transcriptional regulation, even a modest decrease in SMAD4 levels could explain the compromised tumor-suppressive role of TGF-β in HNSCC [41].

The high frequency of *TP53* and *CDKN2A* mutations highlights the key roles of these tumor suppressor genes in HNSCC development. This likely reflects the pre-requirement of their alteration to promote tumor progression and therefore representing one of the earliest events during malignant transformation in HNSCC [48]. The infection with sexually transmitted high risk mucosal HPV provides a molecular mechanism to bypass the need of such alterations, therefore enabling HNSCC development in patients in the absence of carcinogen-induced *TP53* mutations that are characteristic of classical risk factors, such as tobacco and alcohol and betel quid or areca nut chewing [48]. In this regard, it seems paradoxical that most HPV-positive tumors in previous high scale genomic studies [9, 10] were devoid of *TP53* mutations, suggesting that HPV infection precluded the accumulation of *TP53* mutations, or that patients with HPV-associated HNSCC were not exposed to other classical risk factors. In contrast to these possibilities, our analysis in HNSCC cell lines, HNSCC lesions, and recent studies [49] suggest that alteration in *TP53* in HPV^+^ tumors are more frequent than previously expected.

One possible explanation of this discrepancy is that while *TP53* mutations are widespread amongst the tumor mass due to its contribution to initiation and subsequent progression of HNSCC cases associated with classical risk factors, *TP53* mutant cell clones in HPV-associated HNSCC cases may be more limited, in some cases amounting to just 1–10% of the tumor mass. Therefore early sequencing efforts might have been hampered by technological limitations in sequencing depth and mutation calling thresholds could have overlooked this fact. These findings may have direct clinical implications, as HPV^+^ HNSCCs respond better to chemoradiation, leading to a current trend towards dose de-escalation in HPV^+^ HNSCC cases [50]. This therapeutic option may reduce the overall tumor mass with lower undesirable side effects, but patients harboring *TP53* mutations, a typical event in tobacco users [48] concomitant with HPV infection may be at higher risk of tumor relapse after treatment. If this were the case, the prediction that tumor recurrence in HPV^+^ HNSCC cases would involve a higher proportion of tumor cells exhibiting *TP53* mutations could be readily testable in the future, which could directly impact the choice of treatment modality based on the analysis of HPV infection and *TP53* status.

Another emerging observation is that the OPC-22 panel mirrored the absence of prototypical oncogenic drivers in a fraction of HNSCC cases [9, 10, 51], such as in CAL27, WSU-HN6 and WSU-HN8. This suggests that long term culture conditions do not result in the appearance of typical oncogenic drivers [52]. This also raises the possibility of the existence of additional oncogenic mechanisms yet to be characterized in HNSCC. In this regard, epigenetic dysregulation is a key event during cancer development in many tumor types, and epigenetic modifiers consistently rank among the most frequently mutated genes in human cancer [53, 54]. Thus, we can hypothesize that in the absence of other evident oncogenic mechanisms, epigenetic alterations might provide a plausible alternative by abnormally modifying gene expression profiles, resulting in cancer growth.

Specifically, we found a widespread alterations in multiple orthologs of the Drosophila melanogaster Trithorax group (trxG), which are best known for their fundamental role in leukemias [53, 55]. These molecules form part of a multiprotein complex regulating epigenetic events. Among them, MLL1, MLL2 and MLL3 are frequently mutated in HNSCC, together with the H3K27 demethylases KDM6A and KDM6B (UTX and JMJD3, respectively) [55]. Their mutation profile suggests a profound loss of function phenotype. During stem cell fate specification, these molecules are essential for the activation of expression of epigenetically silenced genes [56]. This is also the case for multiple tumor suppressors, which are epigenetically silenced until an oncogenic stimulus provokes their activation, such in the case of the p16 protein product from the *CDKN2A* locus [57]. Aligned with this possibility, all MLL3 mutated cell lines display *CDKN2A* promoter methylation. As cell self-renewal (stemness) maintenance is a hallmark of cancer [58], we can hypothesize that disrupting the function of the trxG complex may represent a driving oncogenic event in HNSCC, as it will interfere with the deployment of tumor suppressive mechanisms, including cell cycle arrest and the initiation of epithelial terminal differentiation programs. These possibilities and the recent development of multiple drugs targeting the epigenetic regulating machinery [59] may provide a rationale for the further exploration of epigenetic modifying agents as alternative targeted therapies in HNSCC, with emphasis on HNSCC cases that do not harbor obvious alterations in driver oncogenic pathways.

Finally, we observed that, despite the diversity of genotypic alterations present in the OPC-22 panel, their gene expression patterns converge into the recurrent dysregulation of a number of gene expression modules that are widely altered in HNSCC, including the widespread dysregulation of multitude of metabolic processes, likely reflecting the metabolic reprogramming recently identified as one of the hallmarks of cancer [58]. In this regard, however, the study of differentially expressed genes in HPV^+^ cell lines may now provide interesting clues on the molecular mechanisms involved in HPV-induced malignancies. Together with well-known oncogenic events induced by the E6/E7 oncoproteins, such as the inactivation of TP53 and the persistent stimulation of E2F transcription factors due to RB1 inhibition [38], the specific HPV^+^ gene expression profiles suggest that other less studied mechanisms might also contribute to HPV-driven HNSCC development. In particular, our transcriptome analysis suggests that HPV oncogenes could regulate both transcription factors MYC and TEAD2, the latter requiring the stimulation of the transcriptional co-activator YAP, both of which can initiate oncogenic signaling and prevention of cell differentiation [60, 61]. HPV-oncogenes my also promote the activation of the MZF1 transcription factor, which harbors tumorigenic potential [62]. On the other hand, we also observed that HPV^+^ HNSCC cells exhibit lower activity of NF-κB/RELA, a pro-survival and –inflammatory transcription factor, which may explain the decreased activation of the innate immune system and increased sensitivity to pro-apoptotic radio- and chemotherapeutic agents in HPV^+^ tumors [63]. Recently, focal deletions on the *TRAF3* gene have been identified in HPV^+^ HNSCC cases [64], which can potentially impact both NF-κB and interferon signaling [65]. We identified *TRAF3* copy loss in the HPV^+^ UPCI:SCC090 cell line, which could partially contribute to the differential gene expression profile displayed by these cells. In addition, our analysis suggests that other additional mechanisms could contribute to HPV-associated malignancies. These include a decreased host antiviral response through impairment of STAT1 function by E6 during the Interferon-α response [66], decreased TGF-β-dependent gene expression leading to an impairment of epithelial differentiation, and the regulation of miRNA138 and miRNA133B, which have been characterized for their tumor suppressive activity in HNSCC [67, 68], thus opening new avenues for future research in HPV-driven cancers.

In summary, HNSCC display a handful of widespread genomic alterations, which can now be evaluated as potential molecular targets for personalized medicine. *TP53* alterations are among the most frequent events in HNSCC, therefore TP53 reactivating molecules could potentially have a wide impact in HNSCC development and progression. Alterations in the *CDKN2A/CDKN2B* locus are also highly frequent. Overactive CDK4/CDK6 after *CDKN2A* loss may be sensitive to newly developed CDK inhibitors [69]. However, as a significant proportion of *CDKN2A/CDKN2B* alterations are due to gene inactivation by promoter and gene methylation, their gene reactivation by the use of small molecules targeting the epigenetic machinery, including histone and DNA methylases and acetylases, could represent an attractive HNSCC management strategy [70]. As described above, this emerging class of mechanism-based therapies could be particularly attractive for HNSCC lesions harboring mutations in epigenetic modifying enzymes but lacking alterations in typical driver oncogenic pathways. In this regard, multiple genomic alterations converging in the activation of the PI3K/AKT/mTOR pathway may explain the overeactivity of this signaling route in most HNSCC [19–21], which provided a rationale for multiple currently open clinical trials targeting PI3K, mTOR, as single agents and as part of combination therapies.

The recent elucidation of the genomic landscape of HNSCC has provided a unique opportunity to understand the molecular basis of HNSCC. In this regard, our current analysis of HNSCC-derived cells have identified multiple alterations underlying the decreased tumor suppressive effect of TGF-β in HNSCC, underscores the presence of *TP53* mutations in a subset of HPV^+^ HNSCC cases, and revealed wide spread mutations and copy number variations in epigenetic modifiers, particularly of the Trithorax gene group. The latter may contribute to HNSCC initiation and progression in a large fraction of HNSCC cases lacking typical oncogenic drivers. Overall, we can conclude that the development of experimental cellular systems reflecting the most frequent oncogenic events in HNSCC together with the identification of novel actionable molecular targets may now facilitate the pre-clinical evaluation of emerging therapeutic modalities for their effectiveness in tumors exhibiting each particular genomic alteration underlying HNSCC progression.

## METHODS

### Cell lines and culture conditions

All cell lines were culture under the same conditions except the ones listed below. HNSCC lines were cultured on DMEM (D-6429, Sigma-Aldrich, St. Louis, MO), 10% fetal bovine serum, 5% CO_2_, at 37°C. UPCI:SCC090, UM-SCC-47 were grown on 50/50 v/v DMEM/F12 media supplemented with 10% FBS. The spontaneously immortalized NOKSI, NOK6 and NOK16 lines were grown on Defined Keratinocyte-SFM (Invitrogen, Carlsbad, CA).

### DNAs

To generate the lentiviral vector pLTiTSA-Smad4-Flag plasmid, the Smad4-Flag cDNA was excised from a pRK5-SMAD4-Flag plasmid [71] using EcoRI/SalI digestion, gel purified and ligated into a pENTR SfiI Shuttle previously linearized with EcoRI/XhoI. Subsequently, the SMAD4-Flag insert was transferred into a Gateway modified pLTiTSA GW (TREtight-GW-IRES-tomato, SV40-rtTA) [72] via Gateway reaction (Invitrogen).

### Antibodies

Antibodies anti EGFR, TGFBR1 and p16 (JC8) were purchased from Santa Cruz Biotechnology (Santa Cruz, CA). Mouse monoclonal Anti-Flag (M2) antibody was purchased from Sigma. Antibodies against PTEN, phospho-AKT^S473^(XP), AKT, phospho-S6, S6, SMAD4, SMAD2, phospho-SMAD2, SMAD3, phospho-SMAD3 and Tubulin-HRP were purchased from Cell Signaling Technology (Beverly, MA). For immunohistochemistry studies, monoclonal mouse anti-human TP53 antibody was purchased from Dako (Carpinteria, CA) and a CINtec p16 staining kit was purchased from Roche Diagnostics (Madison, WI).

### Immunohistochemistry

H&E stained paraffin sections were used for histopathological evaluation. For immunohistochemistry, 5 μm unstained paraffin sections were deparaffinized in 3 changes of SafeClear II (ThermoFisher Scientific, PA, USA), 5 min each, and the hydrated with graded alcohols (100°, 95°, 70°), 2 changes each, 5 min each. The endogenous peroxidase was blocked by incubating for 30 min in 3% H_2_O_2_ in 70° ethanol. Antigens were retrieved with 10 mM citric acid (2.1 g/L) in a microwave oven, 2 min at 100% power, followed by 18 min at 20%. The slides were allowed to cool for 15 min and washed extensively with distilled water, followed by 3 changes of PBS, 5 min each. After blocking with 2.5% BSA in PBS at room temperature, for 30 min, the slides were incubated overnight at 4°C with the appropriate primary antibodies diluted in 2.5% BSA in PBS. The slides were then washed with PBS, 3x for 5 min, and successively incubated biotinylated anti-rabbit/rat immunoglobulins, 1:400 in blocking buffer at room temperature, for minutes, washed with PBS 3x for 5 min each, and incubated with ABC complex (Vector Lab, CA, USA), 30 min at room temperature. The slides were extensively washed with PBS; the reaction was developed with 3, 3′-Diaminobenzidine under microscopic control and stopped with distilled water. The slides were the counterstained with hematoxylin and washed 15 min in running tap water to bluish, dehydrated in graded alcohols (70°, 95°, 100°), cleared in SafeClear II and mounted in Permount mounting media (ThermoFisher Scientific). The histological slides were processed and developed at the same time to minimize inter-assay variability. All stained slides were scanned at 40x using an Aperio CS Scanscope (Aperio, CA, USA) and quantified using the available Aperio algorithms.

### CDKN2A promoter methylation analysis

The methylation status of CDKN2A was determined on bisulfite-treated genomic DNA by a methylation specific PCR method described previously [73].

### HPV detection

The detection of HPV sequences was performed using the LCR-E7 PCR method described by Sasagawa *et al*. [74] based on four pairs of degenerated oligos designed to amplify E6 and the N-terminal part of E7 of most mucosal human papillomaviruses. Amplicons resulting from positive reactions were purified, Sanger-sequenced and analyzed by BLAST search to determine the identities.

### Quantitative PCR

RNA was extracted from exponentially growing cultures by the TriZol method following manufacturer's recommendations (Invitrogen). One microgram total RNA was converted to cDNA using the Superscript III kit (Invitrogen). Quantitative PCR reactions for SMAD7 were run using the PrimeTime SMAD7 Hs.PT.58.39918935 qPCR assay from Integrated DNA Technologies (Coralville, IA). GAPDH was used for normalization, GAPDHfwd-5′-GAAGGTCGGAGTCAACGGATT, GAPDHrev-5′-CGCTCCTGGAAGATGGTGAT.

### Western blotting

Exponentially growing cells were washed in cold PBS, lysed on ice in RIPA buffer (0.5% NaDOC, 0.1% SDS, 25 mM Hepes pH 7.5, 100 mM NaCl, 1.5 mM MgCl_2_, 0.2 mM EDTA, 1% Triton X-100, 20 mM β-glycerophosphate, 0.5 mM DTT, and 2% Halt Protease and Phosphatase Inhibitor Single-Use Cocktail [Thermo Scientific, Rockford, IL, USA]), and cell extracts collected, sonicated, and centrifuged to remove the cellular debris. Supernatants containing the solubilized proteins were quantified using the detergent compatible DC protein estimation kit (Bio-Rad, Hercules, CA); equal amounts by mass were separated by SDS-PAGE, and transferred to PVDF membranes (Millipore Corporation, Billerica MA). Equivalent loading was confirmed with Ponceau-S staining. For immunodetection, membranes were blocked for 1 h at room temperature in 5% non-fat dry milk in T-TBS buffer (50 mM Tris/HCl, pH 7.5, 0.15 M NaCl, 0.1% [v/v] Tween-20), followed by 2h incubation with the appropriate antibodies, in 1% BSA-T-TBS buffer. Detection was conducted by incubating the membranes with horseradish peroxidase–conjugated goat anti-rabbit IgG secondary antibody (Southern Biotech, Birmingham, AL, USA) at a dilution of 1:50,000 in 5% milk-T-TBS buffer, at room temperature for 1 h, and visualized with Immobilon Western Chemiluminescent HRP Substrate (Millipore).

### Proliferation assays

For proliferation assays, cells plated in 24 well plates were incubated with 0.5μCi [^3^H]-thymidine/ml (PerkinElmer, Boston, MA) for the last 4h of the treatment. Cells were washed twice with PBS, and then 3 times with cold 10% trichloroacetic acid for 10 minutes at 4°C. Cells were lysed in 0.5 ml 0.3N NaOH for 1h at 4°C. Samples (250μl) were mixed with 5 ml of scintillation fluid and counted.

### Exome sequencing and RNAseq

Genomic DNA was isolated using the DNAeasy total DNA isolation kit from Qiagen (Valencia, CA). DNA was submitted for sequencing to the NIH Intramural Sequencing Center where it was further processed. Briefly, whole genome libraries with ~280 base inserts and paired-end index adapters were prepared according to Illumina's TruSeq DNA Sample Preparation v2 method. Batches of 24 uniquely barcoded libraries were pooled using equal volumes of input and run on a MiSeq with version 2 chemistry at a loading concentration of 6 pM. The run consisted of 25 cycles followed by an index read. The demultiplexed read counts were used to normalize the DNA input for exome capture where 6 libraries were pooled together. The exome capture was preformed according to Illumina's TruSeq Exome Enrichment Kit protocol. Each captured exome pool was sequenced in 2 lanes on a HiSeq2000 using version 3 chemistry. At least 40 million paired-end 100 base reads were obtained for each sample. Data was processed using RTA version 1.17.20 and CASAVA 1.8.2. For RNAseq samples Poly-A selected mRNA libraries were constructed from 1 μg total RNA using the Illumina TruSeq RNA Sample Prep V2 Kits according to manufacturer's instructions except where noted. The cDNAs were fragmented to ~275 bp using a Covaris E210. Amplification was performed using 8 cycles to minimize the risk of over-amplification. Unique barcode adapters were applied to each library. Libraries were pooled in groups of 7–12 for sequencing. The pooled libraries were sequenced on multiple lanes of a HiSeq2000 using version 3 chemistry to achieve a minimum of 40 million 100 base read pairs. The data was processed using RTA version 1.12.4.2 and CASAVA 1.8.2.

### Data analysis and sample filtering

For DNAseq studies, reads were mapped to the human reference genome (hg19) by the Novoalign aligner. Mapped reads were further processed using the GATK pipeline [75] involving realignment around indels, removal of duplicated reads, base scores recalibration and mutation calling. Multisample VCF files were annotated using the ANNOVAR software [76]. Due to the absence of matching normal DNA for each cell line, we defined a filtering strategy based upon modifications on two recently described approaches to approximate somatic mutations on the NCI-60 cell line panel [77] and the COSMIC Cell Line Project [14], in which somatic mutation calling was approximated by additional filtering rejecting all the non-reference alleles present in the ESP6500 database in order to exclude alleles present in the normal population, except if the allele was present in the COSMIC v64 database [16]. In addition, all the non-reference alleles left after the previous filtering step were discarded if were present in more than 3 cell lines as likely represented putative SNPs not captured in the ESP6500 project, again preserving those alleles present in COSMIC v64. The Strand NGS software (Strand Life Sciences, Bangalore, India) was used to compute copy number variations (CNV) and as a second mutation calling method used to confirm select mutations. For CNV determination, a pseudonormal sample was computed from the average read depths of all the OPC-22 lines and was used to define the copy number baseline against which all the OPC-22 cell lines were compared. Variant effect analysis was performed using the PROVEAN tool [78].

### Transcriptome analysis

For RNA-Seq, the short sequenced reads were mapped to the human reference genome (hg19) by splice junction aligner GSNAP (Genomic Short-read Nucleotide Alignment Program) [79]. We employed several R/Bioconductor packages to accurately calculate the gene expression abundance at whole-genome level using the aligned records (BAM files) and to identify differentially expressed genes (DEGs) between different head and neck cancer cell lines. Briefly, the number of reads mapped to each gene based on the UCSC.hg19.KnownGene database was counted, reported and annotated using the GenomicFeatures, Rsamtools and org.Hs.eg.db packages. To identify differentially expressed genes between H&N cell lines groups (e.g.: normal vs. cancer cell lines), we utilized the edgeR-test based on the normalized number of reads mapped to each gene [80].

Heatmap visualization of differentially expressed transcripts was done with the MultiExperiment Viewer software (v4.9) [81]. For automated functional annotation and gene enrichment analysis, we used the Enrichr online resource [82] and the ClueGO tool [83].

### Statistical Analysis

ANOVA followed by the Tukey *t* test was used to analyze the differences between experimental groups. Data analysis was done with GraphPad Prism version 6.0 for Windows (GraphPad Software, San Diego CA); P values of less than 0.05 were considered statistically significant.

## SUPPLEMENTARY TABLES


