# Supplementary material for: The head and neck cancer cell oncogenome: a platform for the development of precision molecular therapies
Source: Oncotarget. 2014 Nov 4;5(19):8906–23. doi: 10.18632/oncotarget.2417 (PMC4253406; doi:10.18632/oncotarget.2417)
Supplement: Supplementary file 1 [file oncotarget-05-8906-s001.pdf]

SUPPLEMENTARY TABLES

Supplementary Table S1: STR profiles of the OPC-22 panel. Genotyping of the OPC-22 panel by Short Tandem Repeat analysis.

	D3S1358	TH01	D21S11	D18S51	Penta E	D5S818	D13S317	D7S820	D16S539	CSF1PO	Penta D	vWA	D8S1179	TPOX	FGA	AMEL
**93VU147T**	15 17	7 9	30 31	16	11 12	11 12	12	10 11	9 11	11 12	12 13.4	18	13 15	9 11	22	X
**BHY**	17	9	29 31.2	14	14	10	12 13	11 12	12	10	13 14	15 16	11 14	8 11	24	X Y
**BICR22**	15	6 7	28 31	14	12 14	11 12	11 13	11 12	11 12	11	9 13	16 19	10 15	8	24	X
**CAL27**	16	6 9.3	28 29	13	7	11 12	10 11	10	11 12	10 12	9 10	14 17	13 15	8	25	X
**CAL33**	17	9 9.3	29 30	14	7 15	11 12	8 13	8 10	11	11 12	12 13	17	13	8	21 22	X Y
**Detroit 562**	15 16	8 9	28 30	15	13	11 12	12	8 10	11	11 13	13	16	13 14	8 10	21	X
**WSU-HN12**	15	7 9	32 33.2	14	7	11 12	13	10	9	10 11	9 10	16 17	13	11	20 26	X
**WSU-HN13**	14 16	7 9	31.2	14 15	12 14	12	10 12	9 10	12	11	9 11	18	13 15	8 11	21 26	X
**WSU-HN30**	16	7 9	28	13 16	8 12	12 13	11	10	11 12	10 12	2.2	15 18	15 17	6 11	20 24	X Y
**WSU-HN6**	18	6 9	32.2 33.2	13	8 14	11	13	10 11	10 12	11	9	18 19	13 14	8 10	21 25	X Y
**WSU-HN8**	17	6 9	30	13 19	5 13	11 12	13	8	9 12	12 13	15	17 18	11 12	8	22	X Y
**NOKSI**	16	9.3	28 30.2	12	7 12	12	10 12	9 11	9 12	9 11	11 13	16 17	14	11 12	24	X
**ORL-48**	15	6 9	29 32.2	14	7 18	11	12	11	11 13	10	9 11	19	12 15	11	24 25	X
**SCC-15**	16	9 9.3	30 31.2	16	7 13	12	9 14	10 11	12 15	10 13	9 13	15 17	10 13	8	19 24	X Y
**SCC-25**	17	8	30	16	14 15	12	13	12	11 12	10	13	17 19	13	8 12	20 24	X
**UM-SCC-47**	15	7 9.3	29 30	18	12 13	11 12	8 11	11	8 13	11 13	9 10	18	15	10 11	23 25	X Y
**SCC-9**	15	8 9	28	12 14	11	12	9	8	10 11	11	9	17	13	9 11	20 25	X Y
**UPCI:SCC090**	14	7	29 31	14 18	11 12	11 12	11	9 10	12 13	11 12	11	17	12	8	20	X Y
**UD-SCC-2**	14 16	8 9	30 31.2	12 17	10	10 11	8	8 9	11 13	11 12	9 13	15 18	13 15	8 10	20 27	X Y
**UM-SCC-11B**	16	7	28	16	8 13	11	14	11	12 14	7	2.2	16 17 18	15	8 11	19	X
**UM-SCC-17B**	15 18	6 8	28	22	5 19	11	11 13	13	10 11	10 11	10	14 17	12 13	10 11	20 22	X
**UM-SCC-2**	17	6 9.3	30 31.2	15	11	11 13	12	13	9	11	10	14 17	12	8	22 26	X Y
**UM-SCC-4**	16	6	29	16 18	7 10	10 12	12	10 11	9	10 12	11 15	17	14	8 11	17	X

Supplementary Table S2: Associated clinical information of the OPC-22 panel. Available clinical information including age, sex, anatomical location and stage of the OPC-22 cell line panel.

	Age	Sex	Location	Stage
BHY	52	Male	Lower Alveolus	-
BICR 22	88	Male[1]	Tongue/Lymph Node Met	-
CAL27	56	Male[1]	Tongue	-
CAL33	69	Male[1]	Tongue	-
Detroit 562	-	Female	Pharynx/Pleural Effusion Met	-
WSU-HN6	-	Male[2]	Base of tongue	T3N2M0
WSU-HN8	-	Male[2]	Lymph Node	T3N2M0
WSU-HN12	-	Female[2]	Lymph Node Met	T4N1M0
WSU-HN13	-	Female[2]	Tongue	T2N2M0
WSU-HN30	-	Male[2]	Pharynx	T3N0M0
ORL-48	79	Female	Gingiva	T4N2M0
SCC-15	55	Male	Tongue	T4N1M0
SCC-25	70	Male	Tongue	T2N1
SCC-9	25	Male	Tongue	T2N1
UM-SCC-2	64	Female	Alveolar ridge	T2N0M0
UM-SCC-4	47	Female	Base of tongue	T3N2aM0
UM-SCC-11B	65	Male[1]	Supraglotic	T2N2aM0
UM-SCC-17B	47	Female	Soft nissue-neck/Primary in supraglotis	T1N0M0
93VU147T	58	Male[1]	Floor of the mouth	T4N2
UM-SCC-47	53	Male	Oral cavity	T3N1M0
UPCI:SCC090	46	Male	Base of tongue	T2N0
UD-SCC-2	58	Male[1]	Hypopharynx	T1N2M0
NOKSI		Female[2]	Gingiva	-

Supplementary Table S3: *TP53* mutation status of the OPC-22 panel and NOKSI. Description of the mutations detected in *TP53* in the OPC-22 panel and their associated predicted effects.

Cell Line	Mutation	GOF	PROVEAN score	Prediction
BHY	G117fs	-	-	-
BICR 22	G325-I332del	-	−27.00	Deleterious
CAL27	H193L	-	−10.42	Deleterious
CAL33	R175H	Yes	−4.87	Deleterious
Detroit 562	R175H	Yes	−4.87	Deleterious
WSU-HN6	H179L	Yes	−10.42	Deleterious
WSU-HN8	R196X	-	−5.63	Deleterious
WSU-HN-12	-	-		-
WSU-HN13	V173L	Yes	−2.92	Deleterious
WSU-HN30	-	-	-	-
ORL-48	K292fs E336X	-	-	-
SCC-15	-	-	-	-
SCC-25	R209del	-	−7.61	Deleterious
SCC-9	V274_E285del	-	−78.81	Deleterious
UM-SCC-2	R3016_A3017delinsX	-	−5.70	Deleterious
UM-SCC-4	Y126X	-	−7.31	Deleterious
UM-SCC-11B	C242S	-	−9.75	Deleterious
UM-SCC-17B	-	-	-	-
93VU147T	L257R	-	−5.77	Deleterious
UM-SCC-47	-	-	-	-
UPCI:SCC090	-	-	-	-
UD-SCC-2	-	-	-	-
NOKSI	H179Y	Yes	−5.81	Deleterious

GOF: Gain-Of-Function mutation.

Supplementary Table S4: Copy number variations of select epigenetic regulators and *SMAD4* (TCGA). Percentage of homo- and heterozygous deletions present on the TCGA HNSCC provisional dataset.

Gene	% Homozygous Deletion	% Heterozygous Deletion
KDM6A	5.0	29.5
KDM6B	0.7	24.8
EP300	0	15.6
CREBBP	0	11.3
SMAD4	4.6	48.7

Supplemental Data File 1: OPC-22_Exonic_ESP6500filtered.xlsx. Multi-tab Excel file containing all the exonic SNVs detected per cell line excluding synonymous variants and those present on the ESP6500 project.

Supplemental Data File 2: OPC-22PerGeneCNV.xlsx. Excel file containing computed gene copy numbers per gene and cell line. Because of the absence of paired normal computations were conducted against a pseudonormal generated by averaging gene copy values across all the samples and assuming diploidy (2n).

Supplemental Data File 3: OPC-22_RNAseq_DEG.xlsx. Excel file containing the transcriptome of the differentially expressed genes between the OPC-22 panel and three pooled normal immortalized keratinocytes (NOKSI, NOK6, NOK16). Expression values were computed using the DEseq algorithm.

Supplemental Data File 4: OPC-22_Upregulated_TF_enrihment.xlsx. Excel file containing the results of an enrichment analysis against the TRANSFAC and JASPAR databases using the ENRICHR tool on the set of upregulated genes of the OPC-22 cell lines when comparing against normal immortalized keratinocytes (NOKSI, NOK6, NOK16).

Supplemental Data File 5: OPC-22_DEG_HPVvsNONHPV.xlsx. Excel file containing the transcriptome of the differentially expressed genes between HPV^+ and HPV^- cell lines of the OPC-22 panel. Expression values were computed using the DEseq algorithm.

Supplemental Data File 6: OPC-22_ HPVvsNONHPV _TF_ enrichment.xlsx. Multi-tab excel file containing the results of an enrichment analysis against the TRANSFAC and JASPAR databases using the ENRICHR tool on the set of up- and down-regulated genes of the OPC-22 cell lines when comparing HPV^+ vs HPV^- cell lines.
